# The Electron‐Density Distribution of UCl_4_ and Its Topology from X‐ray Diffraction

**DOI:** 10.1002/anie.202413883

**Published:** 2024-11-11

**Authors:** Alessandro Cossard, Christopher G. Gianopoulos, Jacques K. Desmarais, Silvia Casassa, Carlo Gatti, Alessandro Erba, A. Alan Pinkerton

**Affiliations:** ^1^ Dipartimento di Chimica, Università di Torino via Giuria 5 10125 Torino Italy; ^2^ Department of Chemistry School of Green Chemistry and Engineering The University of Toledo Toledo Ohio 43606 United States; ^3^ CNR-SCITEC Istituto di Scienze e Tecnologie Chimiche “Giulio Natta” via C. Golgi 19 20133 Milano Italy

**Keywords:** X-ray diffraction, 5 f electrons, Actinides, Uranium, Quantum theory of atoms in molecules

## Abstract

The chemistry of 5f electrons in actinide complexes and materials is still poorly understood and represents a serious challenge and opportunity for experiment and theory. The study of the electron density distribution of the ground state of such systems through X‐ray diffraction represents a unique opportunity to quantitatively investigate different chemical bonding interactions at once, but was considered “almost impossible” on heavy‐atom systems, until very recently. Here, we present a combined experimental and theoretical investigation of the electron density distribution in UCl__4_ crystals and comparison with the previously reported spin density distribution from polarized neutron diffraction. All approaches provide a consistent picture in terms of electron and spin density distribution, and chemical bond characterization. More importantly, the synergy between experiments and quantum‐mechanical calculations allows to highlight the remarkable sensitivity of X‐ray diffraction to 5f electrons in materials.

## Graphical abstract

We discuss the unprecedented sensitivity of X‐ray diffraction to 5 *f* electrons in actinide materials. This was made possible by recent advances in the experimental setup as well as data reduction strategy, and, crucially, by comparison with accurate quantum‐mechanical simulations. The electron density of UCl_4_ crystals has been analysed. Several indicators have been discussed that reveal the participation of 5 *f* electrons to chemical bonds.

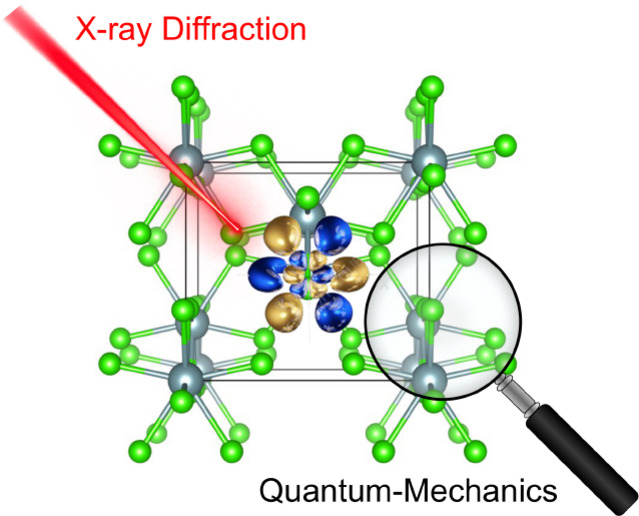

## Introduction

The nature of the *f*‐element (lanthanide, actinide) ligand bond is still poorly understood and presents a serious challenge for both the theoretician and the experimentalist.[^1^, ^2^] In particular, the larger spatial extent of 5 *f* orbitals, relative to 4 *f*, determines a broad valence manifold of 5 *f*, 6*p*, 6*d* and 7 *s* orbitals in actinides, whose hybridization and relative role in the formation of chemical bonds is affected by several factors, and varies along the actinide series.[^3^, ^4^, ^5^, ^6^] In particular, the degree of hybridization and covalency of 5 *f* electrons has received much attention.[^2^, ^3^, ^7^, ^8^, ^9^, ^10^] We note that some level of covalency has been recognized for over 50 years, and many experimental techniques have been applied to characterize the covalent interactions and, in particular, any *f*‐orbital contribution. Shifts in the *f*‐*f* transitions in the uv/vis absorption/fluorescence spectra with different ligands (the nephelauxetic effect, which involves contributions from excited electronic states) has been attributed to covalency involving *f*‐orbitals.[^11^, ^12^, ^13^] The isomer shifts and quadrupolar coupling observed in ^151^Eu and ^237^Np Mössbauer spectra have been interpreted as due to partial transfer of ligand electrons into *f*‐orbitals of the metal.[^14^, ^15^] Photoelectron spectroscopy has been widely used[^16^, ^17^, ^18^] to provide information on the energy of occupied molecular orbitals whose character has then been derived from theory. Although only one experiment to directly determine the *f*‐electron spin delocalization (on UCl_4_) using polarized neutron diffraction has been carried out,^[19]^ magnetic resonance techniques have been used to obtain information on electron spin delocalization, either from ESR/ENDOR spectroscopies,[^20^, ^21^, ^22^] or from contact shifts obtained from NMR.[^23^, ^24^] Nuclear spin‐spin coupling to ^31^P which requires a through bond contact mechanism has been observed for diamagnetic molecules, either to the metal nucleus, J_PY_,^[25]^ (with no possible *f*‐orbital contribution), or through a uranyl center, J_PP_.^[26]^ More recently, NEXAFS studies have provided a new measure of covalency. The intensity observed for largely ligand based transitions may be interpreted in terms of the overlap of filled ligand orbitals with vacant or partially occupied metal orbitals.[^27^, ^28^, ^29^] This has been most successfully applied to the study of the actinide‐chlorine bond via the chlorine 1s→3p transition. The intensity of this transition provides a measure of the percentage of ligand *p* and metal *d* and *f* orbital mixing.

The study of the electron density distribution of the ground state of a crystal through X‐ray diffraction represents a unique opportunity to quantitatively investigate different chemical bonding interactions simultaneously.[^30^, ^31^] This is often complemented by quantum‐mechanical calculations to gain further insight.[^32^, ^33^] The electron density $\rho \left(r\right)$ can be analysed with a variety of techniques, among which the quantum theory of atoms in molecules (QTAIM) provides a robust formal framework within which multiple aspects of chemical bonding can be analyzed.[^34^, ^35^] The theory builds on a topological analysis of the electron density. Accurate experimental investigations of the electron density have shown remarkable agreement with theoretical predictions for typical light‐atom structures, but studies involving heavy‐atom systems were considered “almost impossible” merely three decades ago.^[36]^ However, significant recent advancements in computing, diffraction instrumentation, and experimental methodologies have led to substantial improvements in the field of experimental X‐ray electron density research. Such advances have opened up enticing prospects for the future development of electron density studies on heavy‐atom systems, despite the persisting challenges associated with such experiments.[^37^, ^38^, ^39^, ^40^]

Herein, we report on the synergistic experimental and theoretical investigation of the electron density distribution in UCl_4_ crystals and comparison with the previously reported spin density distribution from polarized neutron diffraction. All approaches provide a very consistent picture in terms of electron and spin density distribution, and chemical bond characterization. More importantly, the combination of experiments and theory allows to highlight the remarkable sensitivity of X‐ray diffraction to 5 *f* electrons in materials.

## Results and Discussion

### Sample Preparation and Structure Characterization

Uranium tetrachloride crystallizes in the tetragonal space group *I*4_1_ */amd*, with four formula units per cell. The asymmetric unit is composed of a uranium atom on a position of D_2d_ point symmetry ($\bar{4}$ 2m, 1/8th site occupancy) and a chlorine atom located on a mirror plane (with 1/2 site occupancy). Its unit‐cell parameters were determined to be **a**=**b**=8.29830(10), **c**=7.4296(2) Å at 110 K with Ag $K_{\alpha }$ radiation (0.56086 Å),^[41]^ falling in between those previously measured by neutron diffraction at 10 K,^[19]^ **a**=**b**=8.312(6), **c**=7.411(6) Å, and at room temperature,^[42]^ **a**=**b**=8.263(3), **c**=7.457(3) Å (we note that this is a rare example of negative thermal expansion). The use of Ag $K_{\alpha }$ radiation provides data of extremely high resolution (up to sinθ/λ =1.68 Å^–1^, *d*=0.298 Å) and reduces the effects of absorption and extinction as compared to longer wavelengths. Hygroscopic, green single crystals of UCl_4_ were afforded by sublimation of a microcrystalline powder in an evacuated quartz tube incubated in a two‐zone tube furnace which was held at 450 and 350 °C for six hours and slowly cooled to room temperature over twelve hours.

**Figure 1:**
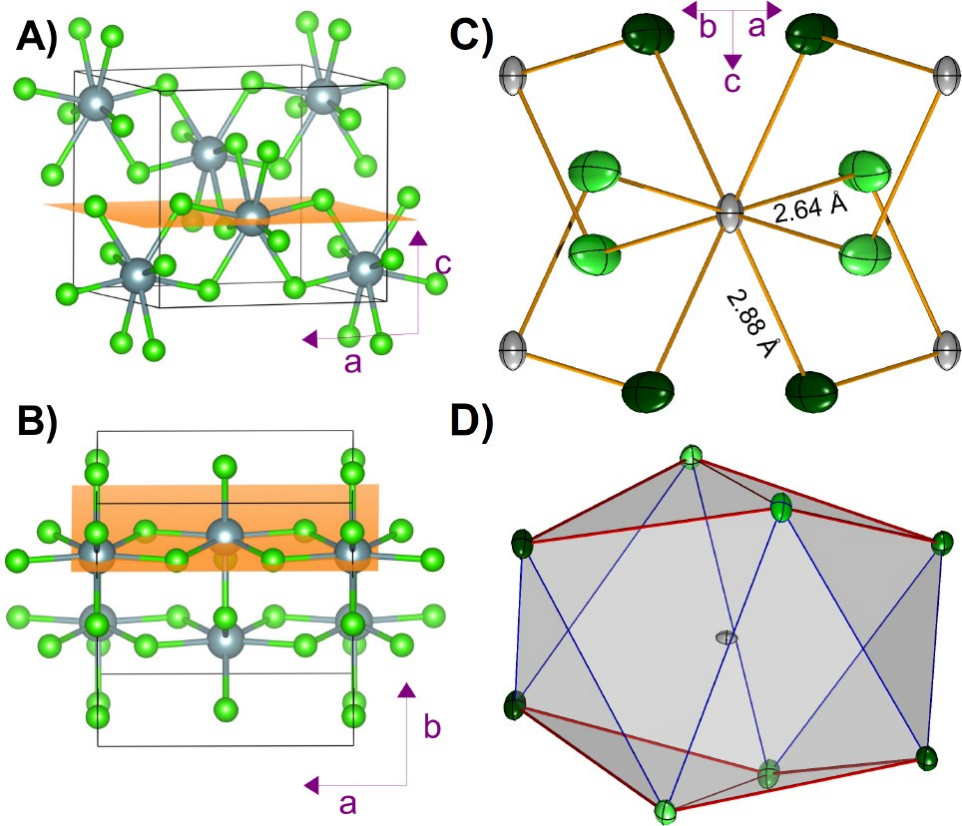
Structure of the UCl_4_ crystal. A−B) Atomic structure of the UCl_4_ crystal with two highlighted crystallographic planes to be considered below. C) ORTEP representation drawn at the 99 % probability level of the atomic structure of a UCl_8_ fragment in the crystal showing the four short (light green) and four long (dark green) U−Cl bonds. D) Depiction of the UCl_8_ disphenoid coordination polyhedron, a.k.a. *D*_2*d*_ dodecahedron. Edges drawn in red are to highlight the distortion from a square antiprism which generates the disphenoid geometry. Light and dark green Cl atoms are shown as in panel C.

The atomic structure of the crystal is shown in Figure 1 A–B). Each U atom is in an 8‐coordinated distorted bisdisphenoid environment (*D*_2*d*_ dodecahedron), with four Cl atoms at a distance of 2.6454(1) Å (hereafter referred to as the “short” U−Cl interactions) and another four Cl atoms at 2.8822(1) Å (hereafter referred to as the “long” U−Cl interactions); Figure 1 C) provides a graphical representation of the spatial orientation of the eight Cl atoms around each U atom in the crystal, with the four Cl involved in the short interactions in light green and the four Cl involved in the long interactions in dark green. The relative strength of the two interactions has recently been investigated computationally via a local vibrational mode analysis.^[43]^ It is known that UCl_4_, ThCl_4_,^[44]^ ThBr_4_,^[45]^ PaCl_4_,^[46]^ PaBr4_4_ ^[47]^ and NpCl_4_ ^[48]^ are all isomorphous but with some slight differences in the ratio of bond lengths for the short and long bonds. All quantum‐mechanical calculations are performed on the experimental structure.

### Spin Density, or Early Signs of 5 f Covalency

As we mentioned in the Introduction, the first ever, direct, evidence of 5 *f* covalency in an Actinide compound dates back to the low temperature (10 K) polarized neutron diffraction measurements by Lander and co‐workers on a UCl_4_ single crystal in 1985.[^19^, ^36^, ^49^] The spin magnetization density of the crystal was measured and compared to that of an ideal ionic model with two unpaired electrons fully hosted by the uranium 5 *f* orbitals. Small but significant differences were reported in the spatial distribution of the unpaired electrons, indicative of a hybridization between the 5 *f* and 6*d* orbitals, with an associated transfer of electrons from 5 *f* to 6*d* orbitals. Their model indicated spin density transfer of about 2 % and was rationalized in terms of partial depletion of the $Y_{0}^{3}\left(r\right)$ orbital (i.e. l=3 , m=0 , or $5f_{z^{3}}$ ) and concomitant partial population of $Y_{\pm 2}^{2}\left(r\right)$ orbitals (i.e. l=2 , m=±2 , or $6d_{x^{2}-y^{2}}$ and 6*d_xy_*).

**Figure 2:**
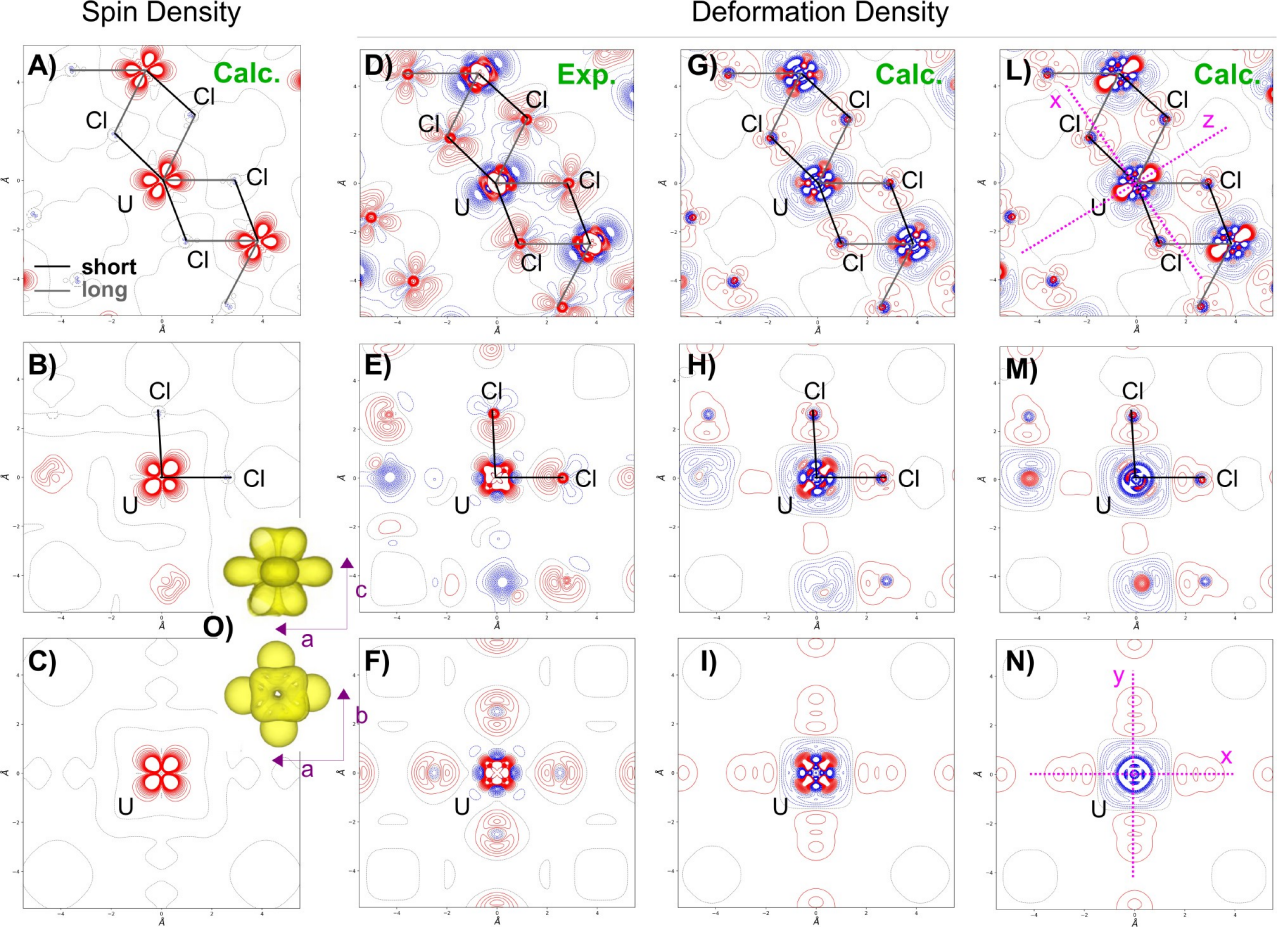
Spin density and deformation density of the UCl_4_ crystal. A−C) Computed spin density *s*(**r**) contour maps on three crystallographic planes. O) 3D view of the computed spin density *s*(**r**) around the U atom in two different orientations. D−F) Experimental deformation‐ density ▵*ρ*(**r**) contour maps. G−I) Same as in D−F) but from theoretical calculations of the ground state. L−N) Same as in G−I) but from a constrained solution with a different population of 5 *f* orbitals (see text). In all contour maps, iso‐valued lines (red for positive, blue for negative) are separated by 0.05 *e/*Å^3^. Spin and deformation density maps are produced with the CRYSTALpytools Python interface to CRYSTAL^[50]^

The spin polarization of the UCl_4_ crystal obtained from our quantum‐mechanical simulations is analysed in Figure 2 A−C,O) and Table 1 in terms of spin density 2D maps, 3D plots, and orbital populations. A simple Mulliken orbital population analysis of the computed electron density $\rho \left(r\right)$ and spin density $s\left(r\right)$ (see Table 1) clearly reveals the presence of two unpaired electrons in the 5 *f* orbitals of U (spin density population of 2.005 $\left|e\right|$ ). A fraction of the spin density of the U atom is also hosted by its 6*d* orbitals (spin density population of 0.047 $\left|e\right|$ ), indicative of 5*f*–6*d* mixing. The spin density population of the 6*d* orbitals amounts to 2.3 % of that of the 5 *f* orbitals, which agrees nicely with the experimental evidence from polarized neutron diffraction.[^19^, ^36^, ^49^] Further corroborating the picture is the analysis of the individual 5 *f* and 6*d* orbital components hosting the spin density (Tables S2 and S3): 99.2 % of the 5 *f* spin density is found on $Y_{\pm 1}^{3}\left(r\right)$ ($5f_{xz^{2}}$ and $5f_{yz^{2}}$ ) and $Y_{\pm 3}^{3}\left(r\right)$ ($5f_{x\left(x^{2}-3y^{2}\right)}$ and $5f_{y\left(y^{2}-3x^{2}\right)}$ ), with $Y_{0}^{3}\left(r\right)$ ($5f_{z^{3}}$ ) contributing with just 0.1 %; at the same time, the largest fraction of the 6*d* spin density (72 %, Table S3) is hosted by $Y_{0}^{2}\left(r\right)$ ($6d_{z^{2}}$ ) and $Y_{\pm 2}^{2}\left(r\right)$ ($6d_{x^{2}-y^{2}}$ and 6*d_xy_*). The spin density on the Cl atoms is very small (0.017 $\left|e\right|$ ), all of which is hosted by 3*p* orbitals, in turn hybridized with the U 6*d* orbitals in the formation of the U−Cl bonds. Overall, the Mulliken spin populations paint a picture of participation of 5 *f* electrons in the bonding, as mediated through coupling with the 6*d* orbitals. Figure 2 A–C) shows 2D contour maps of the computed spin density of the UCl_4_ crystal in three selected planes to analyze its spatial distribution. The plane of the top panel passes through two short and two long U−Cl bonds for each U atom, as shown in Figure 1 B). The plane of the bottom panel is an equatorial plane (perpendicular to the **c** crystallographic axis) passing through U, as shown in Figure 1 A). The plane of the middle panel passes through two short U−Cl bonds. Figure 2 O) shows 3D views of an iso‐surface of the spin density around the U atom in the UCl_4_ crystal.

**Table 1:** Total and spin orbital populations (Mulliken approach) of the UCl_4_ crystal from the computed electron density $\rho \left(r\right)=\rho^{\alpha }\left(r\right)+\rho^{\beta }\left(r\right)$ and spin density $s\left(r\right)=\rho^{\alpha }\left(r\right)-\rho^{\beta }\left(r\right)$ . Total atomic charges Q and spin populations are reported in the last two rows, as obtained from the simple Mulliken approach and the more accurate QTAIM. As stated in the Supporting Information, an all‐electron basis is used for Cl, while the 60 innermost core electrons of U are described by an ECP so that the 32 outermost valence electrons are explicitly treated in the calculations (reference atomic electronic configuration for the valence: $5s^{2}$ $5p^{6}$ 5d^10^ $5f^{3}$ $6s^{2}$ $6p^{6}$ 6d^1^ $7s^{2}$ ).

	U		Cl	
	$\rho \left(r\right)$	$s\left(r\right)$	$\rho \left(r\right)$	$s\left(r\right)$
s	4.063	0.002	5.978	0.000
p	11.934	0.005	11.498	−0.018
d	11.302	0.047	0.036	0.001
f	2.640	2.005	0.000	0.000
g	0.001	0.000		
				
Q_Mulliken_	+2.060	+2.059	−0.512	−0.017
Q_QTAIM_	+2.408	+2.011	−0.602	−0.005
Q_Exp_	+2.040	–	−0.510	–

The picture depicted above is confirmed by the integration of the spin density over the atomic basins of U and Cl within the QTAIM framework, which yields atomic spin density populations of 2.011 and 0.005 $\left|e\right|$ for U and Cl, respectively.

### Electron Density, or the Unprecedented Sensitivity of X‐rays to 5 *f* Electrons

Many peculiar properties of Actinide compounds arise from the much larger spatial extent of their 5 *f* orbitals compared to the 4 *f* orbitals of Lanthanides, for instance. This implies an enhanced propensity of 5 *f* electrons to interact with *p* or *d* electrons of the same atom and neighbouring atoms, producing what is referred to as their hybridization and degree of covalency.[^51^, ^52^]

A detailed analysis of the population of the 5 *f* orbital shell would thus be key to an effective understanding of chemical features of Actinide compounds. Ideally, X‐ray diffraction could represent a direct means to probe the electron distribution around Actinide elements in materials. However, so far the sensitivity of this technique to 5 *f* electrons has proven extremely challenging: “*For example, the spatial distribution of the 92 electrons surrounding the Uranium nucleus can be determined by X‐rays, and the use of tunable synchrotron radiation can alleviate the absorption problem. However, 86 of these electrons are in the Radon core and of little interest. We need to find the spatial distribution of the remaining 6 % to less than 5 %, and clearly this is exceedingly difficult to obtain*”.^[49]^ Here, building on recent technological and data reduction advances,^[40]^ we are going to present clear evidence of the sensitivity of X‐ray diffraction to the specific population of 5 *f* orbitals in UCl_4_. Indeed, current data quality is sufficient to model subtle details of the electron density distribution, even to the point of modelling the populations of diffuse 5 *f* orbitals.

Inspection of Table 1 reveals that, based on our quantum‐mechanical calculations, the electronic configuration of Uranium in UCl_4_ is as follows (compared to its elemental reference [Rn] $5f^{3}$ 6*d* ^1^ $7s^{2}$ ): i) the 7 *s* orbitals are completely depopulated; ii) the 5 *f* orbitals are partially depopulated; iii) the 6*d* orbitals are partially populated; iv) the overall atomic charge is +2.41e from the QTAIM approach (with a corresponding atomic charge of Cl of -0.60e ), which leaves only three to four valence electrons formally associated with U, out of the nominal total of 92 (i.e.  3–4 %). The QTAIM atomic charges from the experimental electron density are in fair agreement with those from theory, with the charge of Cl being -0.51e and the corresponding charge of U being +2.04e . We note that the experimental QTAIM charge of the U atom was derived from the atomic charge of Cl with the assumption of charge neutrality due to convergence not being obtained during basin determination and integration for U despite the use of very fine grid spacing.

Overall, the orbital populations reported in Table 1 draw a picture of a mixed valence system where all orbital types (*p*, *d* and *f*) are actively involved in the formation of chemical bonds. Let us elaborate more on this. When the U atom forms six bonds in an octahedral (or distorted octahedral) coordination environment, as in the [PPh_4_^+^][UF_6_^–^] and Cs_2_UO_2_Cl_4_ crystals which we have studied previously,[^38^, ^39^, ^40^, ^53^, ^54^] the orbital hybridization involved in the formation of the bonds is likely of $sp^{3}d^{2}$ type, thus with no direct participation of *f* electrons that are more easily transferred to the ligands. In the present case, where the U atom forms eight bonds in a disphenoid coordination environment, the orbital hybridization involved in the formation of the bonds is likely of $sp^{3}d^{4}$ type,^[55]^ also suggesting no direct participation of *f* electrons in the formation of the chemical bonds with partial covalent character, at least to a “first‐order” approximation. However, the involvement of *f* orbitals becomes clear due to the transfer of spin‐density from the 5 *f* to 6*d* orbitals. Population analysis also reveals a small and nearly equivalent depopulation of the 6*p* orbitals. Site symmetry considerations on the involvement of *f* orbitals are given in the Supporting Information.

We analyze the spatial distribution of the electrons in the ground state of UCl_4_ in Figure 2 D−I). The six panels report contour maps of the deformation density $\Delta \rho \left(r\right)$ (i.e. difference between the electron density of the actual interacting system and the superposition of atomic, non‐interacting densities, with a neutral atomic reference). Panels D−F) refer to the experimental electron density distribution while panels G−I) refer to the computed ground‐state wavefunction.

The resemblance of the spatial distribution of the deformation density (from theory and experiment) in panels D−I) with that of the spin density reported in panels A−C) strongly suggests that the features revealed by the deformation density mostly arise from populations of 5 *f* orbitals and, on a minor extent, of 6*d* orbitals (i.e. those orbitals that nearly fully host spin density). Indeed, the spin density provides a clean visualization of the 5 *f* electron distribution, while the deformation density provides a visualization of the total charge accumulation and depletion relative to a model consisting of isolated non‐interacting atoms (shown in red and blue, respectively); it is clear that the charge distribution revealed by the deformation density in the vicinity of the Uranium atom is dominated by the rearrangement of its 5 *f* electrons.

In order to corroborate this aspect, we have performed quantum‐mechanical calculations on a constrained wavefunction differing from the ground‐state one just in the population of individual components of the 5 *f* orbital shell (see Table S2 and the associated discussion in the Supporting Information). Deformation density maps from this solution are shown in panels L−N): the spatial distribution of the electron density around each U atom is very different with respect to that from the ground‐state solution of panels G−I) in all explored planes, and is solely due to the different population of the individual 5 *f* orbitals in the two cases. Comparison of panels G−I) and L−N) clearly confirms that the features observed in the deformation density are representative of 5 *f* orbital populations. We report a detailed analysis of the deformation density from the constrained wavefunction, as compared to the ground‐state one, in the Supporting Information.

Going back to the ground‐state solution, the maximally populated 5 *f* components are $Y_{\pm 1}^{3}\left(r\right)$ ($5f_{xz^{2}}$ and $5f_{yz^{2}}$ , with 0.80 *e* each), followed by $Y_{\pm 3}^{3}\left(r\right)$ ($5f_{x\left(x^{2}-3y^{2}\right)}$ and $5f_{y\left(y^{2}-3x^{2}\right)}$ , with 0.42 *e* each), with minor contributions from the other three 5 *f* orbitals, totalling just 0.2 *e*. Red features (i.e. charge accumulation) appear in panels D) and G) with mixed x-z character, reflective of the population of the $Y_{\pm 1}^{3}\left(r\right)$ components (i.e. $5f_{xz^{2}}$ and $5f_{yz^{2}}$ orbitals). On the contrary, on that same plane, a blue feature is observed along the *z* axis, which is consistent with the very low population (just 0.06 *e*) of the $5f_{z^{3}}$ orbital in the computed wavefunction. Also, clear features emerge in the *xy* plane of panels F) and I), reflective of the population of the $Y_{\pm 3}^{3}\left(r\right)$ components (i.e. $5f_{x\left(x^{2}-3y^{2}\right)}$ and $5f_{y\left(y^{2}-3x^{2}\right)}$ orbitals).

Bearing in mind that most of the features of the deformation density of UCl_4_ around the U atom are due to the specific population of 5 *f* orbitals, the agreement of the experiments with theory, although qualitative, is exciting, with the experiments being able to capture most subtle features of the spatial distribution of $\rho \left(r\right)$ around U due to the population of specific 5 *f* orbital components. The depopulation of $5f_{z^{3}}$ and corresponding population of $5f_{xz^{2}}$ and $5f_{yz^{2}}$ are clearly seen in panel D); the population of the $Y_{\pm 3}^{3}\left(r\right)$ ($5f_{x\left(x^{2}-3y^{2}\right)}$ and $5f_{y\left(y^{2}-3x^{2}\right)}$ ) components is clearly revealed in panel F). With this in mind, comparison of both the experimentally determined and computed deformation densities, with the computed spin density is particularly revealing.

The experimental electron density distribution is described by a multipolar expansion around the nuclear positions. It is appropriate to ask whether the expansion is sufficient for *f*‐orbital systems. Clearly the minimum order of the expansion necessary to model *f*‐electron systems is six. As we have previously reported,^[40]^ this is also the maximum value currently allowed in the MoPro system,^[56]^ hence this is a potential limitation in our studies. In order to shed some light on this question, we have compared the theoretical electron density obtained with and without *g*‐type atomic orbitals in the basis functions. As shown in Figure S5, the difference is minimal, being (on average) about three orders of magnitude smaller than the deformation density. Although this does not prove that higher poles in the expansion would not improve the experimental model, it does provide some confidence that the current model is adequate.

Overall, the results discussed above provide compelling evidence of the sensitivity of state‐of‐the‐art X‐ray diffraction measurements to 5 *f* electrons in Actinides (3 electrons out of 92 for U).

### Topology of the Electron Density, or Chemical Bonding in UCl_4_

A robust approach to gain chemical insight from the electron density $\rho \left(r\right)$ of a quantum system is represented by the QTAIM, which stems from its topological analysis. Here, we apply the QTAIM to the theoretical and experimental electron density of UCl_4_. Its topology reveals the presence of two independent bond critical points (BCPs): one along the short U−Cl axis and one along the long U−Cl axis. Table 2 reports the location of such BCPs and various local chemical bond descriptors. Let us analyse first the short interaction. Theory and experiment draw a consistent picture of this U−Cl bond, with the BCP being located at 1.35 Å from U. From inspection of the various descriptors, the short U−Cl bond can be classified as of “incipient covalent” character, having $1<\left|V\right|/G<2$ , a negative total energy density H<0 , (small) positive Laplacian $\nabla^{2}\rho >0$ ), and small and negative bond degree H/ρ .^[57]^ We note that the agreement between theory and experiment on the short bond is beyond qualitative. The tabulated data (Table 2) also permit the comparison of the chemical nature of the short relative to long U−Cl bond in UCl_4_. As expected, the electron density at the BCP of the long bond is significantly lower than that of the short bond (about 60 % from theory and 50 % from experiment). Based on the local bond descriptors, the two chemical interactions are of similar “incipient covalent” type, with the short interaction being more covalent than the long one, as reflected in a lower absolute value of the bond degree H/ρ (more so in the experiment than in the theory).

**Table 2:** Local chemical descriptors of the short and long U−Cl bonds in UCl_4_ derived from the QTAIM analysis of the theoretical and experimental electron density: bond length l (in Å), distances between each of the two atoms involved and the BCP d (in Å), and the local value at the BCP of the electron density ρ (in e/Å^3^), its Laplacian $\nabla^{2}\rho$ (in e/Å^5^), kinetic energy density G (in a.u.), potential energy density V (in a.u.), total energy density H (in a.u.), $\left|V\right|/G$ , and bond degree H/ρ (in a.u.).

	Short U‐Cl			Long U‐Cl	
	Calc.	Exp.		Calc.	Exp.
$l_{U-\mathrm{Cl}}$	2.645	2.645		2.881	2.881
$d_{\mathrm{BCP}-\mathrm{Cl}}$	1.292	1.292		1.391	1.469
$d_{\mathrm{BCP}-U}$	1.353	1.355		1.490	1.419
					
*ρ*	0.466	0.405		0.304	0.189
$\nabla^{2}\rho$	2.868	4.651		1.928	2.338
*G*	0.048	0.058		0.028	0.024
*V*	−0.066	−0.068		−0.036	−0.023
*H*	−0.018	−0.010		−0.008	0.001
$\left|V\right|/G$	1.381	1.173		1.288	0.963
H/ρ	−0.265	−0.169		−0.182	0.036

The analysis of the topology of the electron density $\rho \left(r\right)$ is often complemented by the analysis of its Laplacian $\nabla^{2}\rho \left(r\right)$ or, equivalently, of $L\left(r\right)=-\nabla^{2}\rho \left(r\right)$ , which provides additional information on the spatial distribution of the electrons and in particular on the asphericity of (bonded) atoms.^[58]^ In particular, some critical points of the Laplacian correspond to charge concentrations and depletions in the core and valence shells. Valence shell charge concentrations (VSCCs) are particularly relevant to the rationalization of orbital hybridization and chemical bonding, and can be identified in terms of maxima of *L*(**r**).

**Figure 3:**
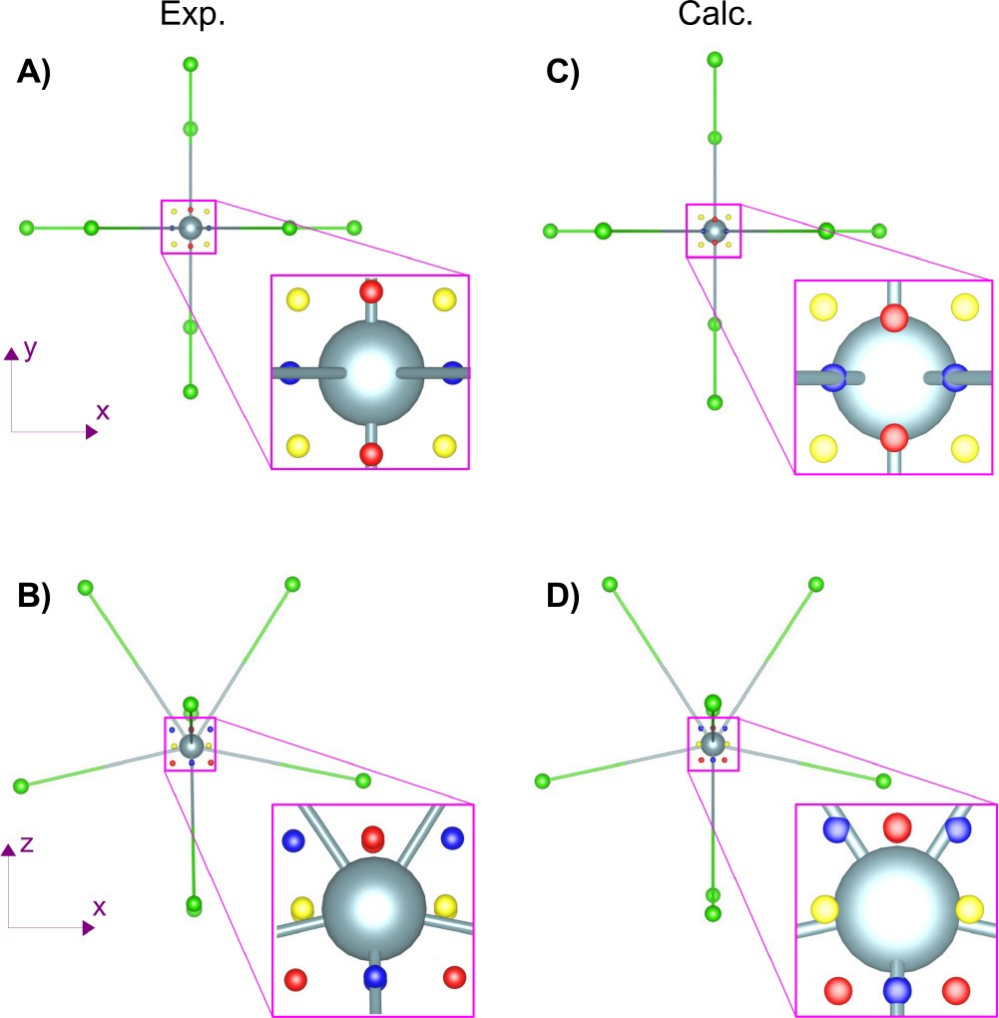
Spatial distribution around the U atom in the UCl_4_ crystal of the twelve VSCC critical points (yellow, blue and red spheres mark three distinct symmetry equivalent sub‐sets): A−B) from the experiment; C−D) from calculations. Top panels present a view down the **c** axis while bottom panels down the **b** axis.

Figure 3 shows the position in space of the outermost VSCCs in the vicinity of the U atom (i.e. those corresponding to the n=6 valence radial region, see Figure 3 in our previous paper:^[53]^) in the UCl_4_ crystal, as derived from a topological analysis of *L*(**r**) from the experiment and from theory. Once more, measurements and calculations provide a very consistent picture. A total of twelve VSCCs are found which, on the basis of symmetry equivalence, are divided into three sub‐sets of four VSCCs each, and with slightly different topological properties. The three sub‐sets are graphically represented by yellow, red and blue spheres. From the theory (and experiment), the four yellow VSCCs form a square on the equatorial *xy* plane, being located along the crystallographic two‐fold axes, and bisecting adjacent (short) Cl ligands. The four blue VSCCs form a tetrahedron and are located almost along the four axes of the long U−Cl bonds. The four red VSCCs also form a tetrahedron, rotated by 90 °C around *z* relative to the blue one, both red and blue VSCCs are found on mirror planes. Only minor distortions from this picture are observed in the experiment, with the blue VSCCs being found a fraction off the axes of the long U−Cl bonds. In the theory, all twelve VSCCs are at a radial distance of 0.30 Å from the U atom while in the experiment they are at a distance of 0.37 Å (yellow set) and 0.38 Å (red and blue sets). Some topological properties of the three sub‐sets of VSCCs are reported in Table S3. They are relatively similar, with the main difference being a larger local value of spin density on the four equatorial ones (yellow), as expected from inspection of Figure 2 A−C). The number, spatial distribution and magnitude of the spin density of such VSCCs is particularly informative. The eight VSCCs which form the red and blue sets are oriented along the U−Cl bonding directions and are consistent with $sp^{3}d^{4}$ type of hybridization.^[55]^ The four VSCCs of the yellow set which are of higher spin density, and directed such that they are “ligand opposed” is consistent with the orbital populations analysed before as well as the transfer of spin density to the 6*d_xy_* orbital.

## Conclusions

In this work, the sensitivity of X‐ray diffraction to 5 *f* electrons in actinide materials has been documented. This was made possible by recent advances in the experimental setup as well as data reduction strategy, and, crucially, by a fine comparison with quantum‐mechanical simulations. In particular, the electron density of UCl_4_ crystals has been analysed, also through its topology, with the quantum theory of atoms in molecules. Several indicators have been discussed that clearly reveal the participation of 5 *f* electrons to chemical bonds through hybridization with 6*p* and 6*d* orbitals.

## Supporting Information

The crystallographic information file (CIF), containing embedded multipole model and experimental structure factors, has been deposited, see Ref. [41]. The authors have cited additional references within the Supporting Information.[^59^, ^60^, ^61^, ^62^, ^63^, ^64^, ^65^, ^66^, ^67^, ^68^, ^69^, ^70^, ^71^, ^72^, ^73^, ^74^, ^75^, ^76^, ^77^, ^78^, ^79^, ^80^, ^81^]

## Conflict of Interests

The authors have no conflicts of interest to declare.

1

## Supporting information

As a service to our authors and readers, this journal provides supporting information supplied by the authors. Such materials are peer reviewed and may be re‐organized for online delivery, but are not copy‐edited or typeset. Technical support issues arising from supporting information (other than missing files) should be addressed to the authors.

Supporting Information

## Data Availability

The data that support the findings of this study are available from the corresponding author upon reasonable request.
