# Supplementary material for: The Electron‐Density Distribution of UCl4 and Its Topology from X‐ray Diffraction
Source: Angew Chem Int Ed Engl. 2024 Nov 11;64(1):e202413883. doi: 10.1002/anie.202413883 (PMC11701361; doi:10.1002/anie.202413883)
Supplement: Supplementary file 1 — Supporting Information [file ANIE-64-e202413883-s001.pdf]

Angewandte Chemie
Eine Zeitschrift der Gesellschaft Deutscher Chemiker

Supporting Information

The Electron-Density Distribution of UCl_4 and Its Topology from X-ray Diffraction

A. Cossard, C. G. Gianopoulos, J. K. Desmarais, S. Casassa, C. Gatti, A. Erba*,
A. A. Pinkerton**

Supporting Information: The Electron-Density Distribution of UCl_4 and Its Topology from X-ray Diffraction

A. Cossard,[†] C.G. Gianopoulos,[*,‡] J.K. Desmarais,[†] S. Casassa,[†] C. Gatti,[¶] A. Erba,[*,†] and A.A. Pinkerton[*,‡]

†*Dipartimento di Chimica, Università di Torino, via Giuria 5, 10125 Torino, Italy*

‡*Department of Chemistry, School of Green Chemistry and Engineering, The University of Toledo, Toledo, Ohio 43606, United States*

¶*CNR-SCITEC, Istituto di Scienze e Tecnologie Chimiche "Giulio Natta", via C. Golgi 19, 20133 Milano, Italy*

E-mail: chris.gianopoulos@utoledo.edu; alessandro.erba@unito.it; a.pinkerton@utoledo.edu

Methods

Experiment

A 0.175×0.140×0.105 mm green crystal of UCl_4, grown by sublimation in an evacuated quartz tube, was mounted on a Rigaku diffractometer with an ULTRAX-18 rotating Ag-anode and a RAPID II image plate detector. The crystal was cooled to 110 K and 547 frames were collected. Indexing and unit-cell refinement were carried out in HKL2000,[S1] intensity integrations were performed in VIIPP[S2,S3] and an absorption correction was applied with CCDABS.[S4] The 97,429 resulting intensities were then corrected for $\lambda/2$ contamination as determined for this dataset[S5] and examined for obvious outliers (e.g. resulting from multiple scattering) following the procedure we have described previously.[S6] Finally, the data were scaled and merged with SORTAV[S7,S8] to give 2,703 independent

reflections for $0 < \sin\theta/\lambda < 1.683$. The 2,262 reflections with $I/\sigma(I)$ ¿ 3 were used for refinement of the structural and total electron density parameters by the modified[S6] Hansen-Coppens multipolar formalism as implemented in the MoPro program suite[S9] with the relativistic wave-function data bank of Volkov and co-workers.[S10] The Coppens Suitability Factor is 0.0165.[S11] Additional details concerning our data reduction methodology and the modified Hansen-Coppens multipolar model employed herein have been reported previously.[S6] A summary of the structure and electron density refinement of UCl_4 is reported in Table S1 of the Supporting Information, along with a discussion of choices made for the refinement. Additional indicators of model quality are also deposited for the spherical atom and multipole models, namely DR plots in Figure S1, scale factor plots in Figures S2 and S3,[S12] and plots of the fractal distribution of the residual density in Figure S4.[S13]

Calculations

Quantum-mechanical calculations are performed within spin density functional theory in a generalized Kohn-Sham framework (through hybridization with exact Fock exchange) with the B3LYP exchange-correlation functional.[S14] All calculations are performed with the Crystal program,[S15–S17] where crystalline orbitals are expressed as linear combinations of atomic orbitals (LCAO).[S18] The topological analysis of the electron density $\rho(\mathbf{r})$ and its Laplacian $\nabla^2\rho(\mathbf{r})$ is performed with the Topond module,[S19,S20] which has recently been improved to efficiently exploit parallel computing,[S21] and to deal with f- and g-type basis functions.[S22,S23] Scalar relativistic effects are included. The effect of spin-orbit coupling (SOC) is also investigated thanks to recent developments in the Crystal software.[S24–S28] Computational details are reported in the Supporting Information.

Quantum Theory of Atoms in Molecules

The quantum theory of atoms in molecules (QTAIM) represents a formal, rigorous technique allowing for a consistent and quantitative description of multiple aspects of chemical bonding through a topological analysis of the electron density $\rho(\mathbf{r})$.[S20,S29] This

requires the evaluation of its first- and second-order derivatives with respect to spatial coordinates in the definition of the gradient vector and Hessian matrix. The trace of the Hessian matrix defines the Laplacian of the density $\nabla^2\rho(\mathbf{r})$. The characterization of chemical bonds passes through the identification of critical points (CPs) of the density, defined as those points in space $\mathbf{r}_{\mathrm{CP}}$ where the gradient vector of the density vanishes $\boldsymbol{\nabla}\rho(\mathbf{r}_{\mathrm{CP}}) = 0$. CPs can be classified into different types according to the signs of the corresponding eigenvalues of the Hessian matrix. First-order saddle points of the density are so-called bond critical points (BCPs). Several local quantities can be evaluated at BCPs, which help characterize the chemical interaction.[S30]

Further information on the spatial distribution of the electrons can be obtained from a topological analysis of the electron density Laplacian, whose negative and positive values correspond to regions of (relative) charge concentration and depletion, respectively.[S31] It is often preferable to flip the sign of the Laplacian and work in terms of the following function:

$$L(\mathbf{r}) = -\nabla^2\rho(\mathbf{r}) \ , \tag{S1}$$

so that positive values and maxima of this function correspond to regions of charge concentration. Critical points of $L(\mathbf{r})$ can be found and characterized by analyzing its first- and second-order derivatives (involving third- and fourth-order derivatives of the electron density, respectively).

Computational Details

Calculations are performed with the CRYSTAL program by use of the global hybrid B3LYP exchange-correlation functional[S14] of the density functional theory (DFT). In the periodic calculations, reciprocal space is sampled on a regular 6×6×6 Monkhorst-Pack grid, corresponding to 68 **k**-points in the symmetry-irreducible Brillouin zone. Scalar relativistic effects are described by use of small-core effective pseudo-potentials, ECP60MDF (with 60 electrons in the core for U).[S32,S33] The valence of U is described by a $(10s8p7d5f1g)/[10s8p7d5f1g]$ basis set: we indicate within round brackets the number

of Gaussian primitive functions used for the various angular quantum numbers and within square brackets the number of shells in which they are contracted. In this case, we use a fully uncontracted basis. With respect to the original basis set optimized for molecular calculations, some very diffuse exponents have been removed (crucially the most diffuse p-type exponent) that were causing linear dependencies in the periodic calculations. Chlorine atoms are described by molecular def2-TZVP basis sets of $(14s9p3d1f)/[5s5p2d1f]$ type, respectively.[S34] SOC is included via a second-variational approach recently implemented in the CRYSTAL software by some of the authors.

Experimental Details

We have described our strategy for expanded Hansen-Coppens refinements for heavy elements [S6] and the application of this strategy to UCl_4 is described in the following. The uranium was split into six pseudo-atoms ($7s$+core, $6d$, $5f$, $6s6p$, $5s5p5d$ and $4s4p4d4f$) while the chlorine atom was treated as a traditional core+valence pseudo-atom. Selection of radial terms for the U pseudo-atom was made in analogy with a small-core ECP with the additional allowance for perturbations of the inner-core n=4 level. Multipole expansion was carried out to the l=6 level for U pseudo-atoms and l=4 for chlorine. The high site symmetry at uranium ($\bar{4}2m$) reduces the number of possible multipoles that can be populated to a total of seven up to the l=6 level. Multipole populations for those directed along the local z-axis were found to be strongly correlated. For this reason, the z-directed multipole for the l=6 level (P6,0) was constrained to zero for all U pseudo-atoms. The multipole expansion was slowly carried out in block refinement steps, with close attention paid to changes in their populations, estimated deviations, improvement of crystallographic agreement factors and by monitoring scale factor plots with the program DRKPlot.[S12] In this way, a 'best' set of multipoles for the split U pseudo-atoms was identified. The Pval populations were intially refined freely during the block refinement stage. After identifying reasonable values, a restraint was applied to prevent divergence during the full least-squares refinement. Aspherical terms were

found not to have a strong impact on the refinement in some cases, especially for the more diffuse terms, namely $7s$ and $6d$. For example, multipoles (excluding Pval) for the U $7s$ pseudo-atom were found to have little influence on the refinement, although in some cases populations would diverge to unrealistic values. Thus, multipoles with l > 1 were constrained to zero for the U 7s pseudo-atom. The same process revealed that two of the seven allowed multipoles improved the quality of the refinement for the U $6d$ pseudo-atom. Similarly, it was determined that inclusion of the P6,0 (z-directed, l=6) multipole did not significantly improve the refinement, while also leading to unrealistically large populations for other z-directed multipoles. As the least-squares surface is relatively flat, restraints were applied as needed to prevent divergence of the full matrix least-squares refinement. A similarity restraint was applied to multipole parameters for the U pseudo-atoms as well as to kappa1 and kappa2 coefficients. A restraint was also applied to kappa2 for the Cl pseudo-atom. In summary, 85 parameters were refined against 2,262 unique, merged reflections with $I/\sigma > 3$ resulting in R1= 0.0069 and wR2 = 0.0131. Unfortunately, it was not possible to obtain a model that would not diverge in the absence of the described restraints. Table S1 reports experimental details on the structure and electron density refinement of UCl_4 crystals.

Table S1: Experimental Details on the structure and electron density refinement of UCl_4 crystals.

Formula	UCl_4
Mr	379.83
Temp of measurement	110.0(1) K
λ (Å)	0.56086
space group	$I4_1/amd$
unit cell dimensions (Å)	a=b=8.29830(10), c=7.4296(2)
V(Å^3), Z	511.616(18), 4
Tmin/Tmax, μ (mm^{-1})	0.325 / 1, 28.431
$(\sin\theta/\lambda)_{max}$ (Å^{-1}), $d_{\max}$ (Å)	1.68, 0.298
reflns integrated	97,429
R_{int}/average multiplicity	0.027, 36.0
independent reflns / (I $>$ 3σ)	2,703 / 2,262
reflns/parameter	26.6
parameters, restraints	85, 22
extinction coeff	0.03965
weighting scheme:[1] a, b	0.001, 0.350
final $R(F)$ all data, $wR2(F^2)$	0.0069, 0.0131
$\Delta\rho_{min}/_{max}$, eÅ^{-3} (all data)	-1.276, 1.286
$\Delta\rho_{min}/_{max}$, ($\sin\theta/\lambda < 1.0$ Å^{-1})	-0.692, 0.632
Coppens Suitability Factor[2] (S)	0.0165

[1] $w = 1 \ / \ \{\sigma^2(F^2) + (ap)^2 + bp\}$, $p = 0.3333F^2_{obs} + 0.6667F^2_{calc}$

[2] $S = V \ / \ \sum_i^{unitcell} n^2_{core,i}$

Supplementary Results (Experiments)

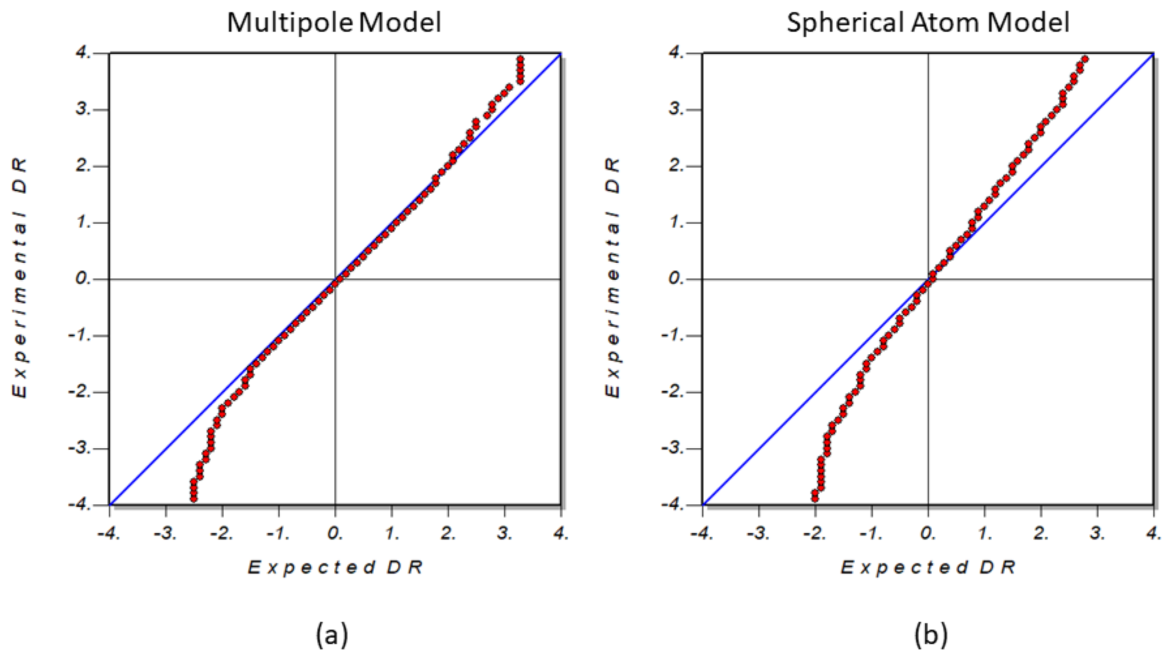

Figure S1: DR plots for the multipole (a) and spherical atom (b) models with identical weighting schemes.

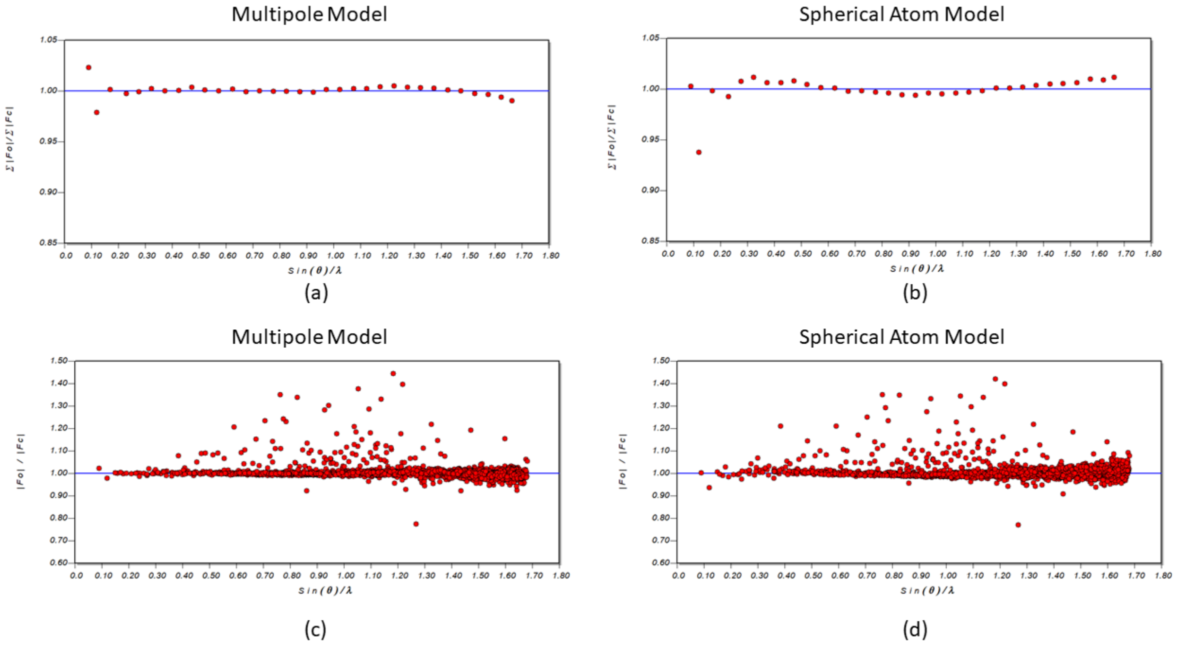

Figure S2: Binned (top panel) and full (bottom panel) scale factor plots against resolution ($\sin\theta/\lambda$) for the multipole (a, c) and spherical atom (b,d) models with identical weighting schemes.

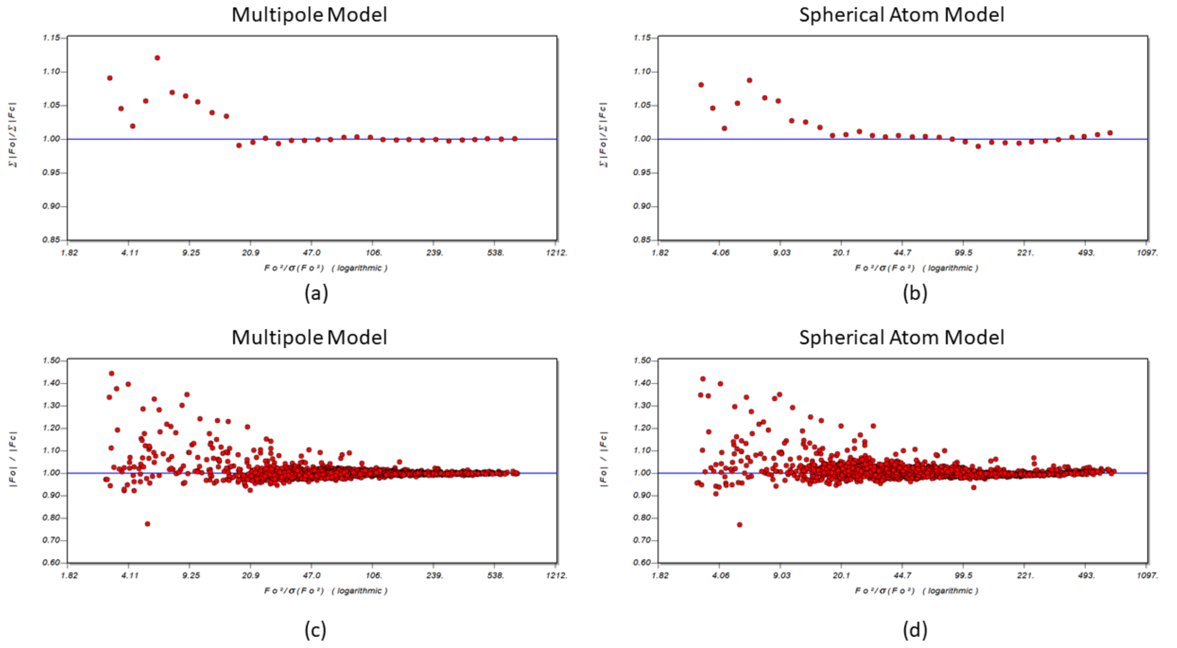

Figure S3: Binned (top panel) and full (bottom panel) scale factor plots against (I/ σ) for the multipole (a, c) and spherical atom (b,d) models with identical weighting schemes.

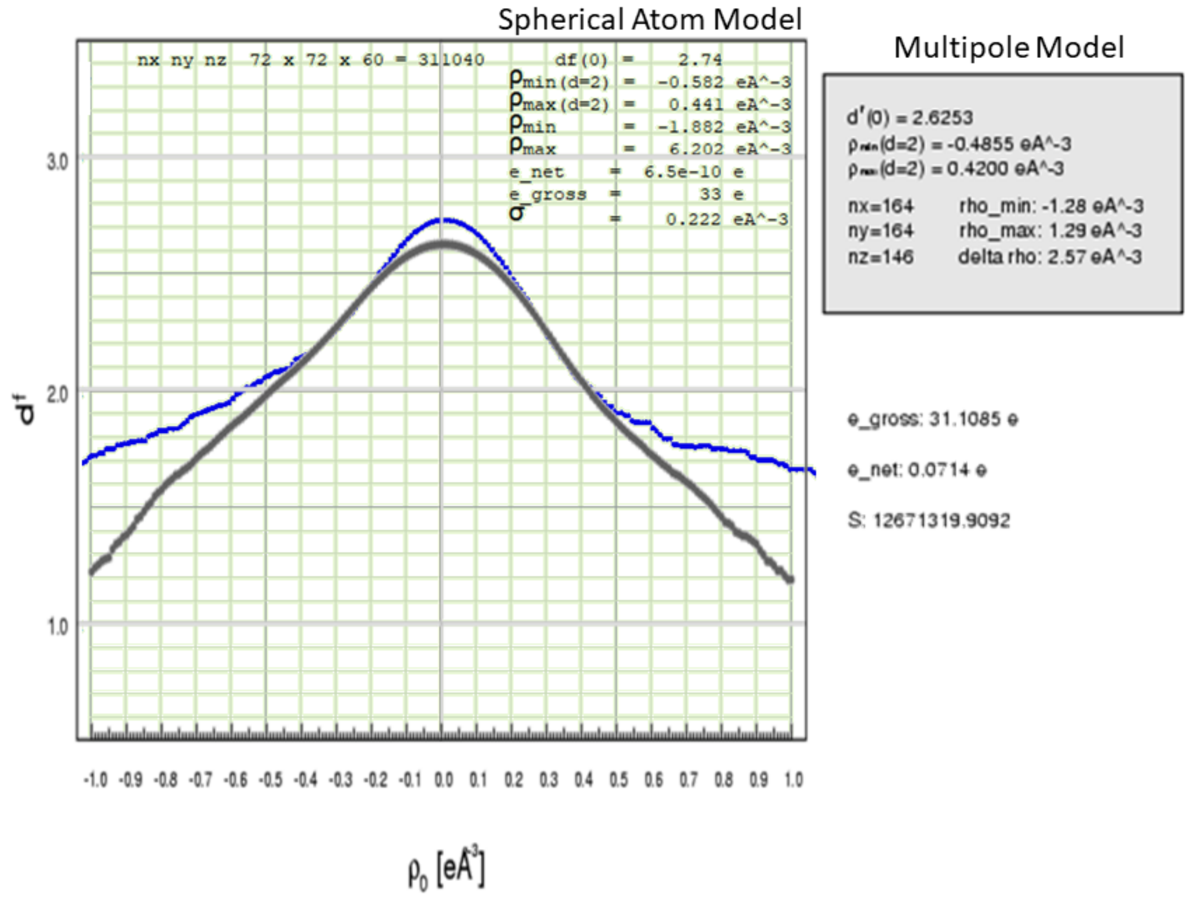

Figure S4: Overlay of fractal dimension distributions of the residual densities for the multipole (grey) and spherical atom (blue) models.[S13]

Supplementary Results (Calculations)

Orbital Populations

The irreducible representations of hybrid orbitals for a variety of coordination geometries given in Table 4 (page 157) of R.B. King, Coordination Chemistry Reviews, 197 (2000) 141-168 only apply to sigma bond formation, and have not considered orbitals higher than d, however, we have f orbitals potentially accessible. For the question of participation of f orbitals in hybrid formation in octahedral geometry, there is a T1u set of f-orbitals available. The D2d dodecahedron can use the A1+B2+2E set of f orbitals as well as the s, p, d orbitals in the King table. Presumably the amount of mixing is a function of the energy match of the various orbitals. This symmetry allowed orbital mixing is in

agreement with the discussion of the small amount of spin density transferred out of the f orbitals.

Table S2: Orbital populations (Mulliken approach) of individual $5f$ components in the computed ground-state wavefunction of the UCl_4 crystal. Values within parentheses refer to a higher-energy constrained wavefunction. The last row reports individual spin-density populations.

	m=0	m=1	m=-1	m=2	m=-2	m=3	m=-3
electron density	0.060	0.796	0.796	0.124	0.028	0.418	0.418
	(1.978)	(0.107)	(0.107)	(0.124)	(0.031)	(0.165)	(0.165)
spin density	0.004	0.683	0.683	0.016	0.003	0.308	0.308

The Constrained Wavefunction

The constrained wavefunction differs from the ground-state wavefunction only in the population of the different components of the $5f$ orbital shell (see Table S2). Indeed, from the simple Mulliken orbital population analysis, in the constrained wavefunction the $Y_0^3(\mathbf{r})$ ($5f_{z^3}$) component is by far the most populated (with about 1.98 e), with the others showing populations < 0.16 e; in the ground-state wavefunction instead the maximally populated components are $Y_{\pm1}^3(\mathbf{r})$ ($5f_{xz^2}$ and $5f_{yz^2}$, with 0.80 e each), followed by $Y_{\pm3}^3(\mathbf{r})$ ($5f_{x(x^2-3y^2)}$ and $5f_{y(y^2-3x^2)}$, with 0.42 e each and minor contributions from the other three f orbitals, totalling 0.2 e).

The most relevant feature in the deformation density from the constrained solution is clearly the electron accumulation along the z axis (i.e. $\mathbf{c}$ lattice vector) visible in panel L) of Figure 2 of the main text and corresponding to the population with 1.98 e of the $Y_0^3(\mathbf{r})$ component (i.e. the so-called $5f_{z^3}$ orbital). In contrast, no particular features are observed in the xy plane in panel N). In the ground-state solution, $5f_{z^3}$ gets depopulated from 1.98 e to 0.06 e and this is clearly seen in the disappearance of the charge concentration (red feature) along z in panel G) relative to L). On the contrary, on that same plane, new features appear with mixed $x - z$ character, reflective of the population of the $Y_{\pm1}^3(\mathbf{r})$ components (i.e. $5f_{xz^2}$ and $5f_{yz^2}$ orbitals). Also, clear features

emerge in the xy plane of panel I) relative to N), reflective of the population of the $Y^3_{\pm 3}(\mathbf{r})$ components (i.e. $5f_{x(x^2-3y^2)}$ and $5f_{y(y^2-3x^2)}$ orbitals).

Table S3: Spin-density populations (Mulliken approach) of individual $6d$ components in the unrestricted computed wavefunction for the ground state of the UCl_4 crystal.

	m=0	m=1	m=-1	m=2	m=-2
spin density	0.012	0.005	0.005	0.007	0.007

Effect of g-type Orbitals

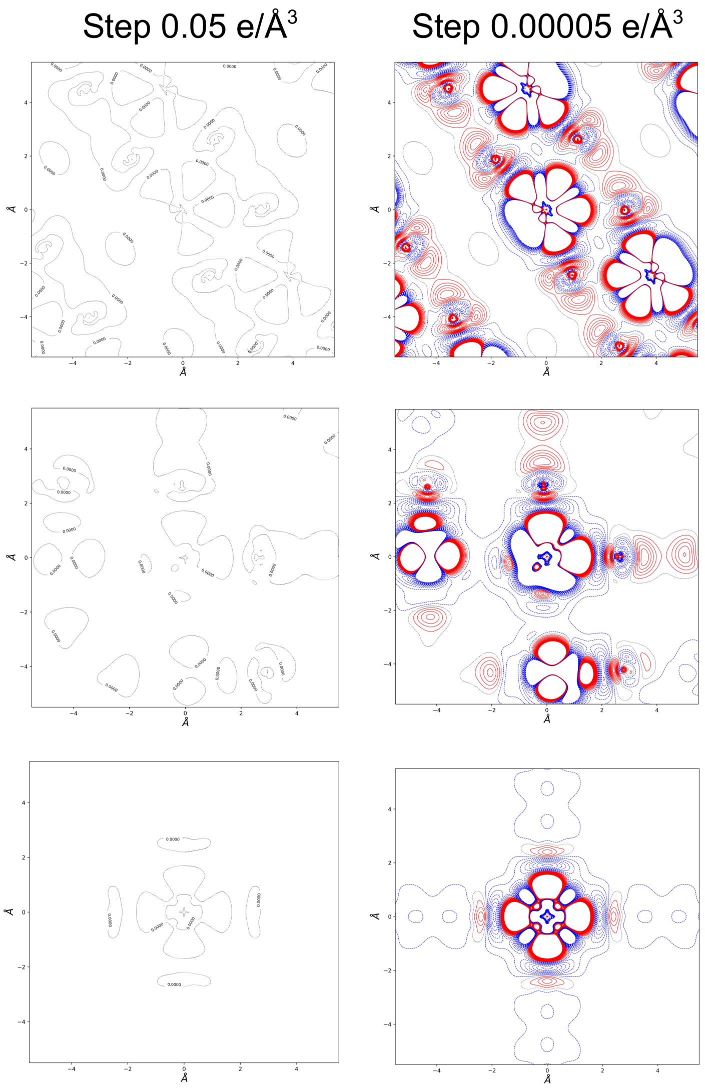

Figure S5: Difference maps of the electron density as simulated with and without g-type atomic orbitals in the basis set of the calculations. (Left panels) Isolines are separated by a step of 0.05 $e/\text{Å}^3$, as in deformation density maps reported in Figure 2 of the main text. (Right panels) Isolines are separated by a step of 0.00005 $e/\text{Å}^3$.

Effect of xc Functional and Structural Relaxation

Table S4: Local chemical descriptors of the short and long U-Cl bonds in UCl_4 derived from the QTAIM analysis of the theoretical electron density from B3LYP and PBE0 xc functionals: bond length l (in Å), distances between each of the two atoms involved and the BCP d (in Å), and the local value at the BCP of the electron density ρ (in $e/\text{Å}^3$), its Laplacian $\nabla^2\rho$ (in $e/\text{Å}^5$), kinetic energy density G (in a.u.), potential energy density V (in a.u.), total energy density H (in a.u.), $|V|/G$ (in a.u.), and bond degree H/ρ (in a.u.).

	Short U-Cl		Long U-Cl	
	B3LYP	PBE0	B3LYP	PBE0
$l_{\mathrm{U-Cl}}$	2.645	2.645	2.881	2.881
$d_{\mathrm{BCP-Cl}}$	1.292	1.293	1.391	1.393
$d_{\mathrm{BCP-U}}$	1.353	1.352	1.490	1.488
ρ	0.466	0.470	0.304	0.304
$\nabla^2\rho$	2.868	2.807	1.928	1.916
G	0.048	0.048	0.028	0.028
V	-0.066	-0.067	-0.036	-0.037
H	-0.018	-0.019	-0.008	-0.009
$\|V\|/G$	1.381	1.396	1.288	1.321
H/ρ	-0.265	-0.273	-0.182	-0.200

Table S5: Local chemical descriptors of the short and long U-Cl bonds in UCl_4 derived from the QTAIM analysis of the theoretical electron density from B3LYP calculations on the experimental geometry (exp-g) and on the fully optimized geometry (opt-g): bond length l (in Å), distances between each of the two atoms involved and the BCP d (in Å), and the local value at the BCP of the electron density ρ (in e/Å^3), its Laplacian $\nabla^2\rho$ (in e/Å^5), kinetic energy density G (in a.u.), potential energy density V (in a.u.), total energy density H (in a.u.), $|V|/G$ (in a.u.), and bond degree H/ρ (in a.u.).

	Short U-Cl		Long U-Cl	
	exp-g	opt-g	exp-g	opt-g
$l_{\mathrm{U-Cl}}$	2.645	2.650	2.881	2.904
$d_{\mathrm{BCP-Cl}}$	1.292	1.295	1.391	1.404
$d_{\mathrm{BCP-U}}$	1.353	1.355	1.490	1.500
ρ	0.466	0.461	0.304	0.286
$\nabla^2\rho$	2.868	2.853	1.928	1.887
G	0.048	0.047	0.028	0.027
V	-0.066	-0.065	-0.036	-0.034
H	-0.018	-0.018	-0.008	-0.007
$\|V\|/G$	1.381	1.383	1.288	1.259
H/ρ	-0.265	-0.263	-0.182	-0.165

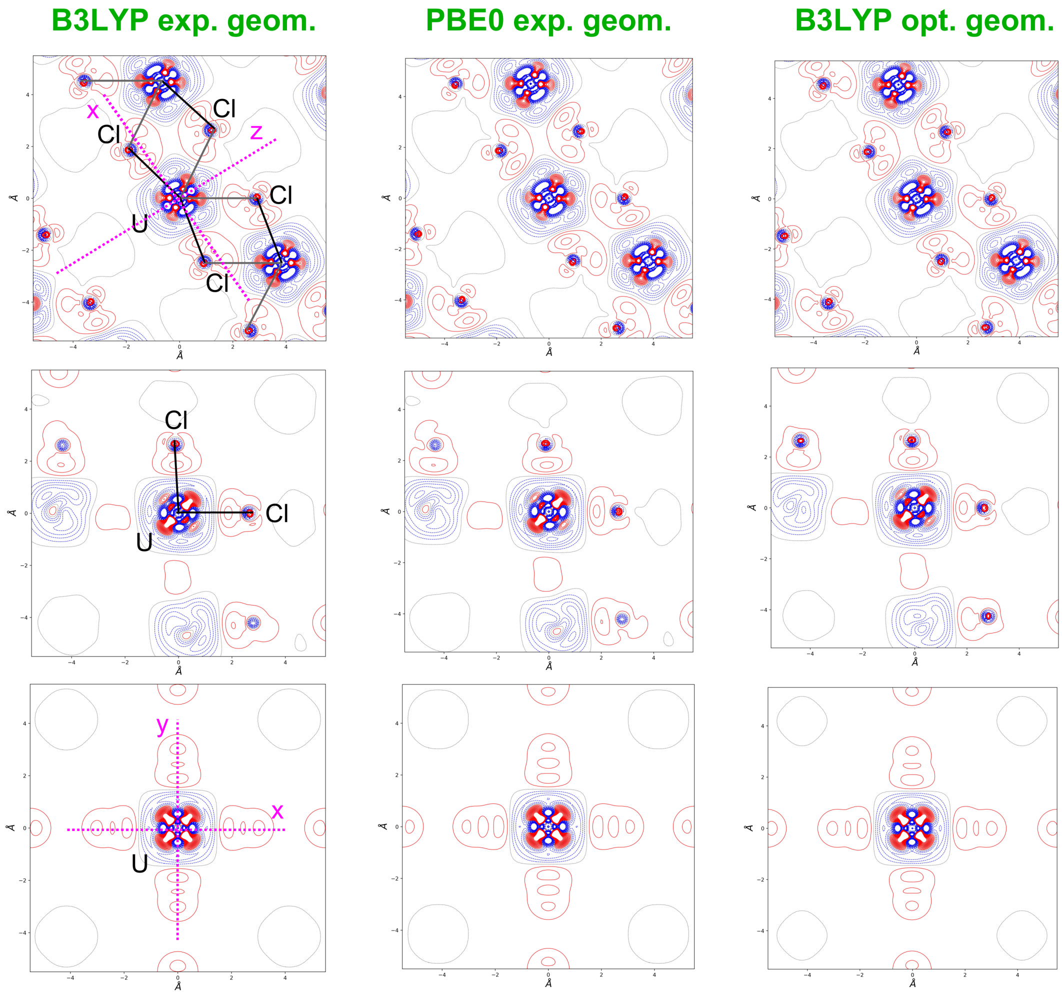

Figure S6: Deformation density maps on the three selected planes from: B3LYP calculations on the experimental geometry (left), PBE0 calculations on the experimental geometry (middle), B3LYP calculations on the fully relaxed geometry (right).

Effect of Spin-Orbit Coupling

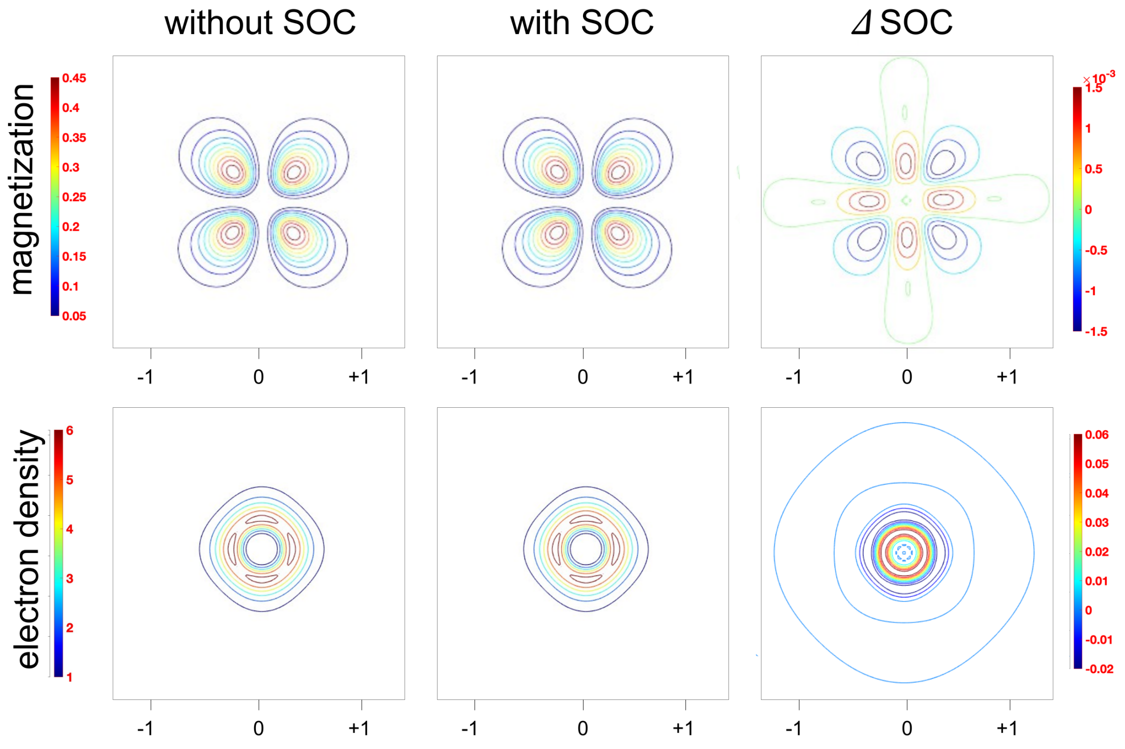

Figure S7: Effect of spin-orbit coupling (SOC) on the magnetization and electron density of UCl_4 in the xy plane passing through the U atom (in the center). The values of the different isolines are given by the color bars (the one to the left for left and center panels, that on the right for the right panel).

Table S6: Local chemical descriptors of the short and long U-Cl bonds in UCl_4 derived from the QTAIM analysis of the theoretical electron density without and with spin-orbit coupling (SOC): bond length l (in Å), distances between each of the two atoms involved and the BCP d (in Å), and the local value at the BCP of the electron density ρ (in $e/Å^3$), its Laplacian $\nabla^2\rho$ (in $e/Å^5$), kinetic energy density G (in a.u.), potential energy density V (in a.u.), total energy density H (in a.u.), $|V|/G$ (in a.u.), and bond degree H/ρ (in a.u.).

	Short U-Cl		Long U-Cl	
	no SOC	with SOC	no SOC	with SOC
$l_{\mathrm{U-Cl}}$	2.645	2.645	2.881	2.881
$d_{\mathrm{BCP-Cl}}$	1.292	1.292	1.391	1.390
$d_{\mathrm{BCP-U}}$	1.353	1.353	1.490	1.491
ρ	0.466	0.466	0.304	0.302
$\nabla^2\rho$	2.868	2.803	1.928	1.911
G	0.048	0.048	0.028	0.028
V	-0.066	-0.066	-0.036	-0.036
H	-0.018	-0.018	-0.008	-0.008
$\|V\|/G$	1.381	1.381	1.288	1.288
H/ρ	-0.265	-0.265	-0.182	-0.179

The Laplacian of the Density

Table S7: Selected topological properties of the twelve VSCCs around the U atom in the UCl_4 crystal, from theory. Local value of L, electron density ρ, spin density s, distance from U, and multiplicity.

Type	$-\nabla^2\rho$ ($e/Å^5$)	ρ ($e/Å^3$)	s ($e/Å^3$)	d_{CP-U} (Å)	Multiplicity
red	4637.198	41.543	1.836	0.30	4
yellow	4759.837	42.170	2.706	0.30	4
blue	4504.800	41.219	1.748	0.30	4

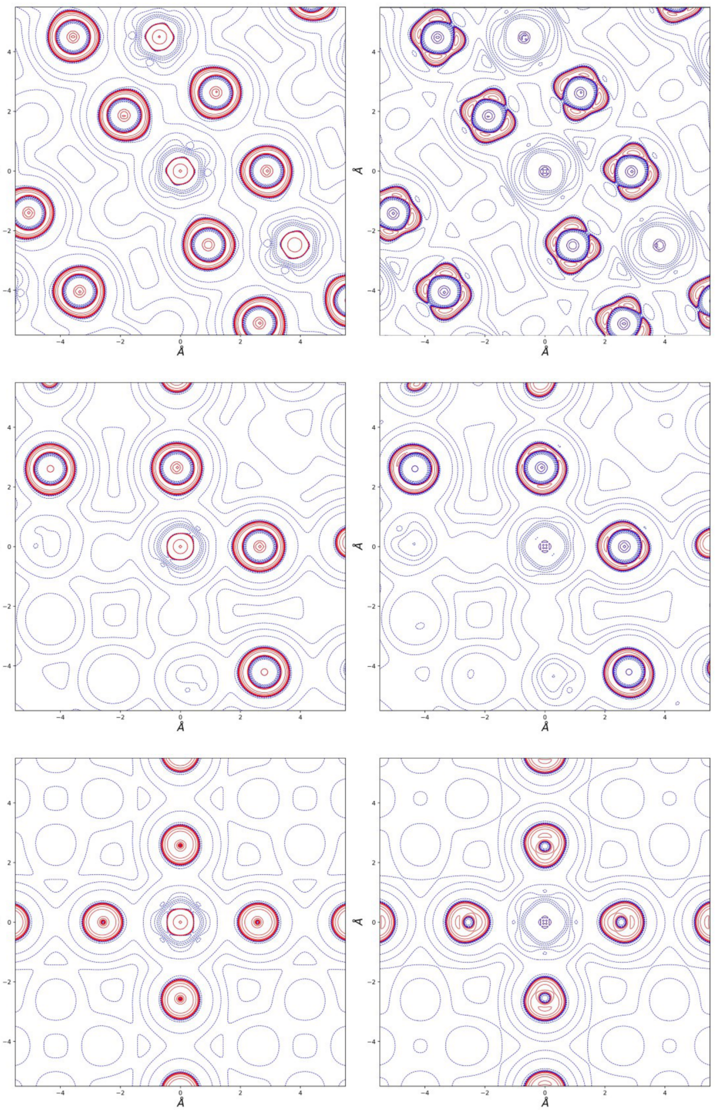

Figure S8: (Left panels) computed and (Right panels) experimental $L(\mathbf{r})$ contour maps of UCl_4 crystals on three crystallographic planes.

References

(S1) Otwinowski, Z.; Minor, W. *Methods Enzymol.*; 1997; Vol. 276; pp 307–326.

(S2) Zhurov, V. V.; Zhurova, E. A.; Pinkerton, A. A. Optimization and evaluation of data quality for charge density studies. *J. Appl. Crystallogr.* **2008**, *41*, 340–349.

(S3) Zhurova, E. A.; Zhurov, V. V.; Tanaka, K. Electron density study of KNiF3 by the vacuum-camera-imaging plate method. *Acta Crystallogr. B* **1999**, *55*, 917–922.

(S4) Zhurov, V.; Tanaka, K. General absorption correction for arbitrary shaped crystal with automatic measurements of crystal shapes. Proceedings of the 28th Japan Workshop of Frontiers of X-Ray Diffraction Technologies in Russia/CIS. 2003; pp 169–178.

(S5) Gianopoulos, C. G.; Chua, Z.; Zhurov, V. V.; Pinkerton, A. A. $\lambda/2$ contamination in X-ray diffraction data–the impact of heavy atoms. *J. Appl. Cryst.* **2017**, *50*, 643–646.

(S6) Gianopoulos, C. G.; Zhurov, V. V.; Pinkerton, A. A. Charge densities in actinide compounds: strategies for data reduction and model building. *IUCrJ* **2019**, *6*, 895–908.

(S7) Blessing, R. H. An empirical correction for absorption anisotropy. *Acta Crystallogr. A* **1995**, *51*, 33–38.

(S8) Blessing, R. H. Outlier treatment in data merging. *J. Appl. Crystallogr.* **1997**, *30*, 421–426.

(S9) Jelsch, C.; Guillot, B.; Lagoutte, A.; Lecomte, C. Advances in protein and small-molecule charge-density refinement methods using MoPro. *J. Appl. Crystallogr.* **2005**, *38*, 38–54.

(S10) Volkov, A.; Macchi, P.; Farrugia, L.; Gatti, C.; Mallinson, P.; Richter, T.; Koritsanszky, T. XD2006-a computer program for multipole refinement, topological analysis

of charge densities and evaluation of intermolecular energies from experimental or theoretical structure factors. *University at Buffalo, State University of New York, Buffalo, New York, USA* **2006**,

(S11) Coppens, P. *X-ray charge densities and chemical bonding*; Oxford University Press, 1997; pp 272–273.

(S12) Stash, A. DRKPlot for XD and SHELXTL. 2007.

(S13) Meindl, K.; Henn, J. Foundations of residual-density analysis. *Acta Crystallogr.* **2008**, *A64*, 404–418.

(S14) Becke, A. D. Density functional thermochemistry. III. The role of exact exchange. *J. Chem. Phys.* **1993**, *98*, 5648–5652.

(S15) Dovesi, R.; Erba, A.; Orlando, R.; Zicovich-Wilson, C. M.; Civalleri, B.; Maschio, L.; Rérat, M.; Casassa, S.; Baima, J.; Salustro, S.; Kirtman, B. Quantum-Mechanical Condensed Matter Simulations with CRYSTAL. *WIREs Comput. Mol. Sci.* **2018**, *8*, e1360.

(S16) Erba, A.; Baima, J.; Bush, I.; Orlando, R.; Dovesi, R. Large Scale Condensed Matter DFT Simulations: Performance and Capabilities of the CRYSTAL Code. *J. Chem. Theory Comput.* **2017**, *13*, 5019–5027.

(S17) Erba, A.; Desmarais, J. K.; Casassa, S.; Civalleri, B.; Doná, L.; Bush, I. J.; Searle, B.; Maschio, L.; Edith-Daga, L.; Cossard, A.; Ribaldone, C.; Ascrizzi, E.; Marana, N. L.; Flament, J.-P.; Kirtman, B. CRYSTAL23: A Program for Computational Solid State Physics and Chemistry. *J. Chem. Theor. Comput.* **2023**, *19*, 6891–6932.

(S18) Desmarais, J.; Erba, A.; Dovesi, R. Generalization of the Periodic LCAO Approach in the CRYSTAL Code to *g*-type Orbitals. *Theor. Chem. Acc.* **2018**, *137*, 28.

(S19) Gatti, C.; Saunders, V.; Roetti, C. Crystal field effects on the topological properties of the electron density in molecular crystals: the case of urea. *J. Chem. Phys.* **1994**, *101*, 10686–10696.

(S20) Gatti, C. Chemical bonding in crystals: new directions. *Z. Kristallogr.* **2005**, *220*, 399–457.

(S21) Casassa, S.; Erba, A.; Baima, J.; Orlando, R. Electron Density Analysis of Large (Molecular and Periodic) Systems: A Parallel Implementation. *J. Comput. Chem.* **2015**, *36*, 1940–1946.

(S22) Cossard, A.; Desmarais, J. K.; Casassa, S.; Gatti, C.; Erba, A. Charge Density Analysis of Actinide Compounds from the Quantum Theory of Atoms in Molecules and Crystals. *J. Phys. Chem. Lett.* **2021**, *12*, 1862–1868.

(S23) Cossard, A.; Casassa, S.; Gatti, C.; Desmarais, J. K.; Erba, A. Topology of the Electron Density and of Its Laplacian from Periodic LCAO Calculations on f-Electron Materials: The Case of Cesium Uranyl Chloride. *Molecules* **2021**, *26*, 4227.

(S24) Desmarais, J. K.; Flament, J.-P.; Erba, A. Spin-orbit coupling from a two-component self-consistent approach. I. Generalized Hartree-Fock theory. *J. Chem. Phys.* **2019**, *151*, 074107.

(S25) Desmarais, J. K.; Flament, J.-P.; Erba, A. Spin-orbit coupling in periodic systems with broken time-reversal symmetry: Formal and computational aspects. *Phys. Rev. B* **2020**, *101*, 235142.

(S26) Desmarais, J. K.; Komorovsky, S.; Flament, J.-P.; Erba, A. Spin–orbit coupling from a two-component self-consistent approach. II. Non-collinear density functional theories. *J. Chem. Phys.* **2021**, *154*, 204110.

(S27) Desmarais, J. K.; Boccuni, A.; Flament, J.-P.; Kirtman, B.; Erba, A. Perturbation Theory Treatment of Spin–Orbit Coupling. III: Coupled Perturbed Method for Solids. *J. Chem. Theor. Comput.* **2023**, *19*, 1853–1863.

(S28) Desmarais, J. K.; Erba, A.; Flament, J.-P. Structural relaxation of materials with spin-orbit coupling: Analytical forces in spin-current DFT. *Phys. Rev. B* **2023**, *108*, 134108.

(S29) Bader, R. F.; Nguyen-Dang, T. *Advances in Quantum Chemistry*; 1981; Vol. 14; pp 63–124.

(S30) Espinosa, E.; Alkorta, I.; Elguero, J.; Molins, E. From weak to strong interactions: A comprehensive analysis of the topological and energetic properties of the electron density distribution involving X–H... F–Y systems. *J. Chem. Phys.* **2002**, *117*, 5529–5542.

(S31) Popelier, P. On the full topology of the Laplacian of the electron density. *Coord. Chem. Rev.* **2000**, *197*, 169–189.

(S32) Cao, X.; Dolg, M.; Stoll, H. Valence basis sets for relativistic energy-consistent small-core actinide pseudopotentials. *J. Chem. Phys.* **2003**, *118*, 487–496.

(S33) Dolg, M.; Cao, X. Accurate relativistic small-core pseudopotentials for actinides. Energy adjustment for uranium and first applications to uranium hydride. *J. Phys. Chem. A* **2009**, *113*, 12573–12581.

(S34) Weigend, F.; Ahlrichs, R. Balanced basis sets of split valence, triple zeta valence and quadruple zeta valence quality for H to Rn: Design and assessment of accuracy. *Phys. Chem. Chem. Phys.* **2005**, *7*, 3297–3305.
